# The Effect of Preparatory Posture on Goalkeeper's Diving Save Performance in Football

**DOI:** 10.3389/fspor.2019.00015

**Published:** 2019-08-21

**Authors:** Rony Ibrahim, Idsart Kingma, Vosse de Boode, Gert S. Faber, Jaap H. van Dieën

**Affiliations:** ^1^Department of Human Movement Sciences, Faculty of Behavioural and Movement Sciences, Vrije Universiteit Amsterdam, Amsterdam Movement Science, Amsterdam, Netherlands; ^2^Adidas miCoach Performance Centre, AFC Ajax, Amsterdam, Netherlands

**Keywords:** biomechanics, stance width, knee angle, center of mass, sports coaching

## Abstract

Identifying the optimal preparatory posture of football goalkeepers can be very relevant for improving goalkeepers' diving save performance, and coaching practices of technical and strength and conditioning coaches. This study aimed to analyse the effect of different starting stance widths and knee flexion angles on movement time, center of mass (CoM) trajectory and velocity in goalkeepers' diving saves. Ten elite goalkeepers performed dives from preferred (PT) and imposed postures, by altering knee angle (45, 75, and 90°) and stance width (50, 75, and 100% of leg length) independently, at the starting position. Repeated measures ANOVA showed a main effect of preparatory posture on dive time (*p* < 0.01). Pairwise comparisons showed that the fastest dive movement time was observed when goalkeepers started from a stance width of 75% (SW75). CoM traveled a larger distance between contralateral and ipsilateral peak ground reaction forces in SW75 than PT (*p* < 0.05). The goalkeepers were also more efficient in SW75, as a smaller countermovement and vertical velocity range were observed during high and low dives, respectively, from SW75 than PT (*p* < 0.05). Thus, diving from a position with wider stance width than the preferred one leads to shorter movement time, and a faster and more direct CoM trajectory toward the ball.

## Introduction

When athletes prepare to perform an explosive movement, they set themselves in a specific preparatory posture, often referred to as the power position (Chiu, 2007). This position is assumed to allow optimal development of joint torques to generate the appropriate motor response to a stimulus (Slater-Hammel, 1953; Loockerman, 1973; Hopkins, 1984). The goalkeeper's diving save in football, especially during set plays (i.e., penalty shot, direct free kick), is one of those explosive skills where the athletes have sufficient time to set themselves in what is assumed to be the optimal preparatory posture before reacting and executing the dive. The goalkeeper's technical coach is usually responsible for teaching and perfecting this preparatory posture for the diving save. In addition, characteristics of this posture are used interchangeably by technical and strength and conditioning (S&C) coaches to make the training, on the field and in the gym, as specific to the goalkeepers as possible (Sheppard et al., 2016). Ibrahim et al. (2019) found that the preferred preparatory posture of elite goalkeepers was characterized by a stance width (SW) of 33% of leg length, 62° knee flexion angle (KA), and 63° hip flexion angle. The effect of starting KA and SW on vertical jump performance was studied before, aiming to find the optimal preparatory posture for the maximal vertical impulse (Martin and Stull, 1969; Domire and Challis, 2007; Gheller et al., 2015). However, what is applicable to vertical jump may not be applied to the diving save, as the two skills are relatively independent of one another (Meylan et al., 2009). For example, the optimal SW for vertical jumps may be too narrow for developing large horizontal sideward forces (Martin and Stull, 1969). Additionally, in the vertical jump the athlete's aim is to propel the body vertically in the air (unidirectional simultaneous push-offs), whereas the goalkeeper's diving save is often characterized by multidirectional sequential push-offs (one leg after the other).

KA and/or SW in the preparatory posture were also varied and studied in other sports skills, such as in tennis and field hockey, showing that the preferred position adopted by athletes may not be always the most optimal one for performance (Loockerman, 1973; Hopkins, 1984; Yamamoto, 1996; Ball and Giblin, 2009). No study to date has explicitly examined the influence of preparatory posture variables, such as KA and SW, on goalkeeper's diving save performance in football. Previous studies established the path and/or the velocity of the goalkeeper's center of mass (CoM) as key performance indicators (Suzuki et al., 1987; Spratford et al., 2009). In addition, the development of a large horizontal (lateral) linear velocity was found to be more critical than the vertical velocity (Ibrahim et al., 2019). Also, the contralateral leg (the leg opposite to the diving side) was found to be the main contributor to the CoM horizontal (lateral) linear velocity when diving toward the corners (Ibrahim et al., 2019). We expected that standing wider than the previously identified preferred SW in diving saves (33% of leg length; Ibrahim et al., 2019) and vertical jumps (5–10 inches; Martin and Stull, 1969), would make the contralateral leg better oriented, for development of horizontal lateral ground reaction forces.

The aim of this study was to independently vary the SW and KA in the preparatory posture of elite goalkeepers and to analyse the effect on diving save performance (i.e., movement time). In addition, we aimed to assess the effect of preparatory postures on CoM trajectory and velocity. We hypothesized that wider than preferred SW would lead to faster dives, and greater CoM horizontal velocity toward the corners.

## Materials and Methods

Ten elite males football goalkeepers (age 18.4 ± 1.6 years, mass 82.6 ± 5.6 kg, height 186 ± 1.5 cm) participated in this study. All participants, or their parents, provided informed consent and the research protocol was approved by the Ethics Committee of the Faculty of Behavioral and Movement Sciences of the Vrije Universiteit Amsterdam. The participants' level, at the time of the experiment, was as follows: two goalkeepers in the Dutch Eredevisie (the highest level of competition nationally), six goalkeepers in the Dutch Eerste Divisie (the second highest level of competition nationally), and two goalkeepers in the Dutch under-17 Eredevisie (the highest level of competition nationally for players under 17 years of age). The experiments were conducted at the Adidas miCoach Performance Center of AFC Ajax. Participants had not suffered any injury that prevented them from performing the diving save at their maximum power or that caused them to change their movement pattern at the time of the experiment.

### Data Collection and Pre-processing

The experimental set-up, data collection and pre-processing are described in detail elsewhere (Ibrahim et al., 2019). Briefly, trajectory data from 44 markers attached to the goalkeepers, were captured using 10 infrared cameras at 200 Hz (Vicon 612, Oxford, UK) while diving to save high (190 cm from force plate level) and low (30 cm from force plate level) balls. Goalkeepers reacted to a visual stimulus produced by a light-emitting diode board placed at the penalty spot, consisting of four lamps indicating the side and height of the ball to save. A ball was suspended 1 m in front of the goal line (with a small magnet attached to a thin rope) at low height on one side, while the second ball was suspended at high height on the other side. After each dive, the ball was re-attached to the rope to avoid any anticipation of height and side. Ground reaction forces (GRF) produced by each leg were measured separately at a rate of 1,000 Hz with two custom-made strain-gauge based force plates (Vrije Universiteit Amsterdam, Amsterdam, The Netherlands). In addition to measuring goalkeepers' diving saves from their own preferred preparatory postures, they also dived from imposed preparatory postures. KA was manipulated during the preparatory posture using a goniometer (0° for upright standing) and by imposing three different conditions: 45° (KA45), 75° (KA75), and 90° (KA90). SW was also manipulated during the preparatory posture, by normalizing SW to personal leg length and imposing three different conditions with feet positions indicated with tape on the force plates: 50% leg length (SW50), 75% leg length (SW75), and 100% of leg length (SW100). When KA was manipulated, SW was left to the goalkeepers' choice, and vice-versa.

Before starting the measurement, goalkeepers performed a specific warm-up with their coaches and around eight diving saves to get familiar with the experimental set-up. The experiment protocol was performed in the following order: (1) five successful dives using goalkeepers' own preferred posture (PT1), randomized over side and height of the dive, (2) 24 successful dives in total, randomized over side, height and posture (KA45, KA75, KA90, SW50, SW75, SW100), and (3) five successful dives using goalkeepers' own preferred posture (PT2), randomized over side and height of the dive. Section (3) of the experimental protocol was included to take potential learning effects during the measurements into account. The goalkeepers performed a total of 34 dives, four dives per preparatory posture (one dive per height, side, and posture), with 1-min recovery time between dives. Only during PT1 and PT2, five trials were measured instead of four in order to prevent any anticipation of the height and side of the last trial for each preferred posture. During the imposed postures, full order randomization allowed only four trials to be performed. A dive was considered successful if: (1) an immediate response to the light, and (2) a dive to the correct side and height, (3) finishing with a hit or catch of the ball was observed. Trials were repeated if one of these conditions was not met, and/or when the goalkeeper indicated that his performance in the dive was not good.

### Data Analysis

All kinematic and kinetic analyses were carried out using custom software in MATLAB (R2015b, MathWorks Inc., US). Six time instants were identified for the diving save: Light signal, dive onset, contralateral peak force (CPF), ipsilateral peak force (IPF), take-off, and ball contact.

#### Definition of Timing Variables

Methods for detecting the dive onset have been described in detail elsewhere (Ibrahim et al., 2019). Briefly, we detected the dive onset using an algorithm based on the Approximated Generalized Likelihood-ratio (AGLR) (Staude and Wolf, 1999). It works by (1) detecting the alarm time (the time instant when the signal reaches the pre-set threshold) using a sliding test window, then (2) tracking back the signal to detect the initial change time using Maximum Likelihood techniques (Poor, 1988). We used a threshold equal to 20% of goalkeeper bodyweight, and three different input signals: total horizontal GRF, total vertical GRF and vertical GRF of the contralateral leg (the leg opposite to the diving side). The dive onset was defined as the average of the two out of three onset estimates, having the smallest mutual difference.

All timing variables were defined relative to the onset of movement. The instant of CPF and IPF, were defined as the instants that the contralateral and ipsilateral leg exerted their maximum resultant GRF, respectively. Contralateral and ipsilateral push-offs time periods were considered as 10 samples prior to 10 samples after CPF and IPF, respectively. Take-off was defined as the instant that the vertical component of GRF, summed over legs, dropped below the 10% bodyweight threshold and ball contact as the instant that a shift in position of the ball's markers was detected in any direction.

To express performance, total dive time was calculated as the time from light signal to ball contact, and split into two main parts: reaction time and movement time. Reaction time was calculated from light signal to the detected dive onset, and movement time was from dive onset to ball contact.

#### Kinematic Analysis

SW was calculated as the distance between the medial malleoli and was expressed as a percentage of the participant's leg length. The leg length of each goalkeeper was measured from the palpated greater trochanter to the ground while the participant was standing bare feet. KA was defined as the Euler angle of the shank anatomical coordinate system (ACS) relative to the thigh ACS. The sequence of decomposition was: flexion-extension, external-internal rotation and abduction-adduction (Wu et al., 2002).

Instantaneous position and velocity of goalkeepers' CoM, in the vertical and horizontal (lateral) directions, were calculated to explain possible differences in diving save performance. The positions and velocities of CoM were analyzed from the light signal to the end of the fastest dive.

### Statistical Analysis

All time series, except CoM position and velocity, were time-normalized from the light signal until ball contact. All data are presented as mean ± standard error between participants. For all tests, the level of significance was set at *p* < 0.05, and the effect size measure partial eta-squared (0.01 small, 0.06 medium, 0.14 large) and Cohen's d (0.2 small, 0.5 medium, 0.8 large) were reported. The assumption of normality was checked by visual inspection of the q–q plots, the box plots and by the Shapiro-Wilk test. There were no violations of these assumptions. All statistical analyses were carried out using IBM SPSS Statistics 20.

#### Dive Times and Reaction Times

Dive times and reaction times were compared between preparatory postures (eight levels: PT1, SW50, SW75, SW100, KA45, KA75, KA90, PT2), diving heights (high and low) and sides (right and left) with three-way repeated measures ANOVAs. After finding no effect of side on any of the variables, all data were averaged over sides and two-way repeated measures ANOVAs (preparatory posture and diving height) were performed and presented in this paper. This was done to simplify the results and focus on the main question. In case of significant effects of preparatory posture, planned comparisons were performed to find significant differences between instructed postures vs. PT1 and vs. PT2. Subsequently, the posture that led to the shortest movement time was compared to all others.

#### Preparatory Postures

Differences in preparatory postures (SW and KA) were tested between PT1 and PT2 with paired *t*-tests, to test for any learning effect.

#### CoM Trajectories and Velocities

The nature of differences between PT1, PT2, and the condition that resulted in the fastest dive was explored in more detail. To this end, the following one-way repeated measures ANOVAs, had preparatory posture (3 levels: PT1, PT2, and the condition that resulted in the fastest dive) as the only factor. Differences in the vertical and horizontal distances traveled by the CoM were statistically analyzed with one-way repeated measures ANOVA, per height and for three time periods (from dive onset to CPF, from dive onset to IPF, and between CPF and IPF). In addition, differences in the average vertical and horizontal CoM velocities during contralateral push-off (10 samples prior to 10 samples after CPF) and during ipsilateral push-off (10 samples prior to 10 samples after IPF) were statistically analyzed, per height, with one-way repeated measures ANOVA. Finally, differences in the countermovement range (CoM position at dive onset—CoM at the lowest position before CPF) during high dives, and in the vertical CoM velocity range (peak positive—peak negative CoM vertical velocity before CPF) during low dives, were statistically analyzed with one-way repeated measures ANOVA. These comparisons helped to interpret any possible physical and/or technical differences in diving save performance between postures.

## Results

### Dive Times and Reaction Times

**Figure 1:**
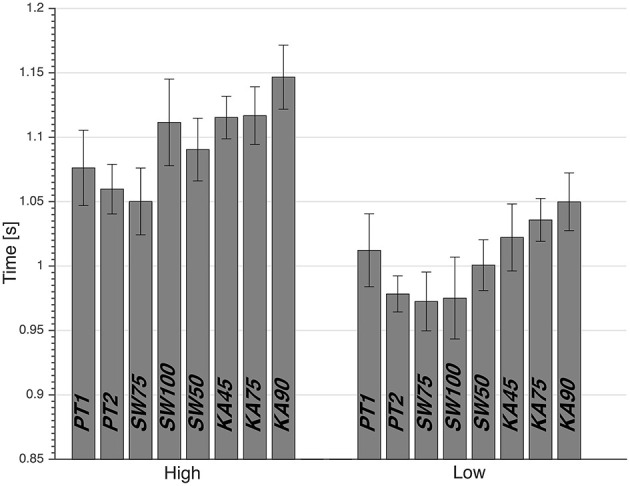
Average dive time, with standard error, from calculated movement onset to detected ball contact. PT1, preferred preparatory posture before the imposed postures' trials; PT2, preferred preparatory posture after the imposed postures' trials; SW50, stance width equal to 50% leg length; SW75, stance width equal to 75% leg length; SW100, stance width equal to 100% leg length; KA45, knee flexion angle of 45°; KA75, knee flexion angle of 75°; and KA90, knee flexion angle of 90°.

Two-way repeated measures ANOVA showed that there were no significant effects of preparatory posture (*p* = 0.68) and height (*p* = 0.84) on reaction times. However, it did show a main effect of height and preparatory posture on dive times (*p* < 0.01), with large effect sizes of 0.43 and 0.97, respectively, and with no interaction effect (*p* = 0.12). Low dives were significantly faster than high dives (*p* < 0.01). PT1 was significantly faster than KA90 (*p* < 0.05), significantly slower than SW75 (*p* < 0.05), and not significantly different from KA45 (*p* = 0.10), KA75 (*p* = 0.11), SW50 (*p* = 0.93), and SW100 (*p* = 0.96; Table 1). PT2 was significantly faster than KA45 (*p* < 0.05), KA75 (*p* < 0.01) and KA90 (*p* < 0.01), and was not significantly different from SW50 (*p* = 0.15), SW100 (*p* = 0.45), and SW75 (*p* = 0.69; Table 1). The SW75 condition resulted in the fastest dive with an average of 1.050 s for high balls and 0.972 s for low balls (Figure 1). Pairwise comparisons showed that SW75 was significantly faster than all the other conditions (*p* < 0.05), except PT2 that showed to be not significantly different with a tendency to be slower than SW75 (Table 2). Based on the statistical results of dive times, all dives from the imposed postures except SW75 were similar or slower than dives from preferred posture (PT1 and PT2). Thus, the rest of the paper will focus on analyzing and explaining differences in performance between SW75 and the preferred posture (PT1 and PT2), separately for high and low dives.

**Table 1:** Planned comparison of dive times averaged over heights, of all imposed postures vs. PT1 and vs. PT2 (^*^p < 0.05).

**Posture compared to PT1/PT2**	**PT1—addressed posture Mean difference (95% CI)**	**PT2—addressed posture Mean difference (95% CI)**
SW100	+0.001 s (−0.037, 0.039)	−0.024 s (−0.094, 0.046)
SW75	+0.033 s^*^ (0.001, 0.065)	+0.008 s (−0.037, 0.052)
SW50	−0.001 s (−0.034, 0.031)	−0.027 s (−0.065, 0.011)
KA45	−0.025 s (−0.055, 0.006)	−0.05 s^*^ (−0.095, −0.005)
KA75	−0.032 s (−0.074, 0.010)	−0.057 s^*^ (−0.087, −0.027)
KA90	−0.054 s^*^ (−0.098, −0.010)	−0.079 s^*^ (−0.128, −0.031)

*PT1, preferred preparatory posture before the imposed postures' trials; PT2, preferred preparatory posture after the imposed postures' trials; SW50, stance width equal to 50% leg length; SW75, stance width equal to 75% leg length; SW100, stance width equal to 100% leg length; KA45, knee flexion angle of 45°; KA75, knee flexion angle of 75°; and KA90, knee flexion angle of 90°*.

**Table 2:** Pairwise comparisons of dive times across postures, after averaging over dive heights.

**Posture compared to SW75**	**Mean difference (SW75—addressed posture)**	**95% confidence interval**		**Cohen's d**	***P*-value**
PT1	−0.033 s	−0.065	−0.001	0.79	**0.044**
PT2	−0.008 s	−0.052	+0.037	0.14	0.699
SW100	−0.032 s	−0.062	−0.002	0.82	**0.038**
SW50	−0.034 s	−0.053	−0.016	1.42	**0.003**
KA45	−0.057 s	−0.085	−0.030	1.58	**0.001**
KA75	−0.065 s	−0.099	−0.031	1.44	**0.002**
KA90	−0.087 s	−0.122	−0.052	1.93	**0.001**

*P-values in bold are significant. PT1, preferred preparatory posture before the imposed postures' trials; PT2, preferred preparatory posture after the imposed postures' trials; SW50, stance width equal to 50% leg length; SW75, stance width equal to 75% leg length; SW100, stance width equal to 100% leg length; KA45, knee flexion angle of 45°; KA75, knee flexion angle of 75°; and KA90, knee flexion angle of 90°*.

### Preparatory Postures

**Figure 2:**
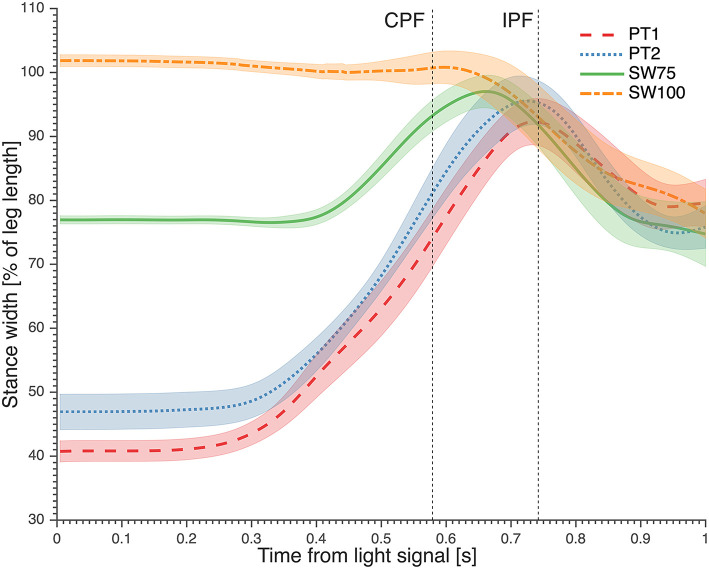
The average stance width kinematics from light signal. Contralateral peak force (CPF) and ipsilateral peak force (IPF) are marked. Trial of stance width equal to 100% leg length (SW100) was added to show that goalkeepers reduced their stance width when starting from this position. PT1, preferred preparatory posture before the imposed postures' trials; PT2, preferred preparatory posture after the imposed postures' trials; SW75, stance width equal to 75% leg length.

Paired *t*-tests between PT1 and PT2 showed that during PT2 participants adopted a SW that was significantly wider than during PT1 (+6% of leg length (*p* < 0.01), with 95% confidence of intervals of 39–42% and 44–50% of leg length for PT1 and PT2, respectively; Figure 2). SW75, the fastest preparatory posture, had an actual starting SW of 76.9 ± 1% of leg length (Figure 2).

### CoM Trajectory and Velocity for High Dives

**Figure 3:**
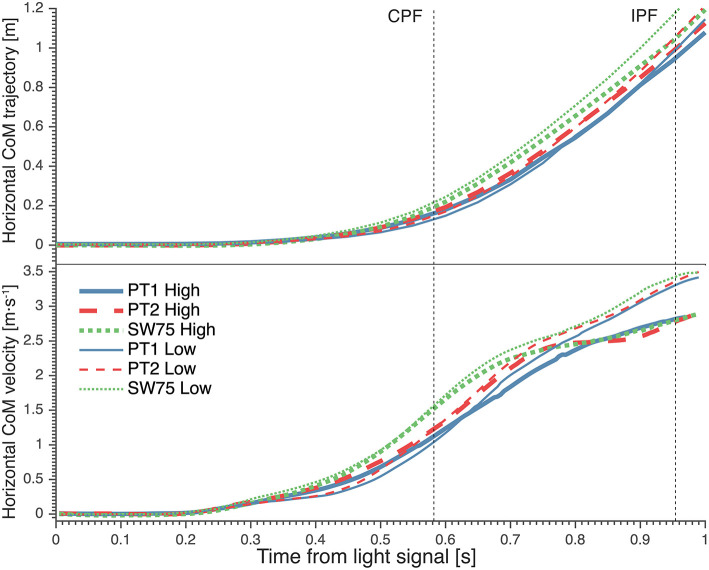
The average horizontal trajectory **(Top)** and horizontal velocity **(Bottom)** of goalkeepers' center of mass (CoM) from light signal during high (thick lines) and low dives (thin lines). The vertical dotted lines indicate the moment of contralateral peak force (CPF) and ipsilateral peak force (IPF). PT1, preferred preparatory posture before the imposed postures' trials; PT2, preferred preparatory posture after the imposed postures' trials; SW75, stance width equal to 75% leg length.

**Figure 4:**
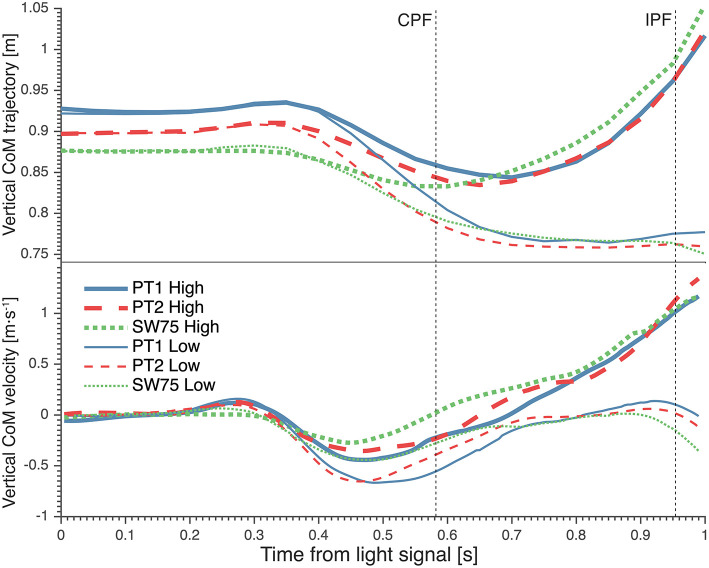
The average vertical trajectory **(Top)** and vertical velocity **(Bottom)** of goalkeepers' center of mass (CoM) from light signal during high (thick lines) and low dives (thin lines). The vertical dotted lines indicate the moment of contralateral peak force (CPF) and ipsilateral peak force (IPF). PT1, preferred preparatory posture before the imposed postures' trials; PT2, preferred preparatory posture after the imposed postures' trials; SW75, stance width equal to 75% leg length.

During high dives, the CoM traveled over a larger horizontal distance, between CPF and IPF (Figure 3; Table 3), in SW75 than PT1 (+9.17 cm; *p* < 0.01) and PT2 (+5.22 cm; *p* < 0.01). Furthermore, the CoM traveled over a significantly larger vertical distance, between CPF and IPF (Figure 4; Table 3), in SW75 than PT1 (+4.94 cm; *p* < 0.05) and PT2 (+5.31 cm; *p* < 0.01). In addition, the goalkeepers performed smaller countermovement (Figure 4; Table 3) during SW75 than PT1 (−5.89 cm; *p* < 0.01) and PT2 (−3.39 cm; *p* < 0.05).

**Table 3:** Follow-up one-way repeated measures ANOVAs of center of mass (CoM) trajectory and velocity (section CoM trajectories and velocities) with preparatory posture [three levels: preferred preparatory posture before the imposed postures' trials (PT1), preferred preparatory posture after the imposed postures' trials (PT2), and stance width equal to 75% leg length (SW75) as a factor.

	**Variables**	**PT1**	**PT2**	**SW75**	***P*-value (partial eta^**2**^)**
High dives	Horizontal distance traveled by CoM between CPF and IPF	59.55 cm	63.50 cm	68.72 cm	0.001 (0.58)
	Vertical distance traveled by CoM between CPF and IPF	10.25 cm	9.88 cm	15.19 cm	0.009 (0.49)
	Countermovement range	14.11 cm	11.61 cm	8.22 cm	0.001 (0.58)
	Average horizontal CoM velocity at contralateral push-off	1.07 m·s^−1^	1.17 m·s^−1^	1.43 m·s^−1^	0.003 (0.52)
	Average vertical CoM velocity at contralateral push-off	−0.27 m·s^−1^	−0.25 m·s^−1^	0.01 m·s^−1^	0.006 (0.52)
Low dives	Horizontal distance traveled by CoM from dive onset to CPF	20.95 cm	23.94 cm	32.84 cm	0.004 (0.51)
	Vertical distance traveled by CoM from dive onset to IPF	18.04 cm	15.94 cm	12.39 cm	0.008 (0.45)
	Average horizontal CoM velocity at contralateral push-off	0.59 m·s^−1^	0.68 m·s^−1^	0.96 m·s^−1^	0.005 (0.49)
	Vertical CoM velocity range	0.92 m·s^−1^	0.88 m·s^−1^	0.72 m·s^−1^	0.02 (0.385)

*Only the tests with a significant result are reported in this table. CPF, contralateral peak force; IPF, ipsilateral peak force*.

These differences in CoM trajectories were manifested in significant differences in average CoM velocities. The average horizontal velocity of CoM during contralateral push-off (Figure 3; Table 3) was significantly greater in SW75 than in PT1 (+0.36 m·s^−1^; *p* < 0.01) and PT2 (+0.26 m·s^−1^; *p* < 0.05). Furthermore, the average vertical velocity of CoM during contralateral push-off (Figure 4; Table 3) was significantly greater in SW75 than in PT1 (+0.28 m·s^−1^; *p* < 0.05) and PT2 (+0.26 m·s^−1^; *p* < 0.01).

### CoM Trajectory and Velocity for Low Dives

During low dives, the CoM traveled over significantly greater horizontal distance, from dive onset to CPF (Figure 3; Table 3) in SW75 than in PT1 (+11.89 cm; *p* < 0.01) and PT2 (+8.90 cm; *p* < 0.05). In addition, the CoM traveled less vertical distance, from dive onset to IPF (Figure 4; Table 3) in SW75 than in PT1 (−5.65 cm; *p* < 0.05) and PT2 (−3.55 cm; *p* < 0.05). These differences in CoM trajectory during low dives were manifested in the average horizontal CoM velocity at contralateral push-off (Figure 3; Table 3), which was significantly greater in SW75 than in PT1 (+0.37 m·s^−1^; *p* < 0.01) and PT2 (+0.28 m·s^−1^; *p* < 0.05). In addition, the vertical CoM velocity range (Figure 4; Table 3) was less pronounced in SW75 than in PT1 (−0.20 m·s^−1^; *p* < 0.05) and PT2 (−0.16 m·s^−1^; *p* < 0.05).

## Discussion

In accordance with our hypothesis, the goalkeepers dived faster and contacted the ball earlier when starting with a SW wider than their preferred one, while KA was similar to their preferred one. The optimal starting position, resulting in the fastest dive, was when SW was set to 75% of personal leg length and KA was ~55°. Goalkeepers were free to adopt their preferred KA during the trials where SW changes were imposed and during PT1 and PT2. For all these trials, KA was similar to the preferred KA found in this study (58 ± 3°). All the modifications to the starting position were unfamiliar to the goalkeepers, thus the significant improvement relative to PT1 is of interest when taking into consideration the amount of hours spent training and performing using their preferred postures. The conditions with instructed KA at the starting position all led to increased movement times. The KA conditions (KA45, KA75, KA90) were significantly slower than all the other conditions. Modifying SW to SW75 did lead to a better performance that was significantly faster than all the other conditions, except PT2. A possible reason for the latter result is a learning effect that may have occurred toward the end of the experiment, as the SW at the starting position during PT2 was significantly greater than PT1. We can distinguish two aspects of learning that may have occurred in this study. The first is learning to improve performance in the experimental set-up; we accounted for it by randomization and by again measuring preferred posture trials at the end of the protocol. While the second is choosing to change the preparatory posture in PT2 trials after being exposed to other imposed postures. This has probably occurred, but it could not affect the imposed postures as these were completely randomized. SW50 was randomized with SW75 and with other imposed postures. Additionally, SW50 was similar to PT2 and close to PT1 in terms of preparatory posture (SW and KA), and showed to be significantly slower than SW75 (*p* < 0.01).

When diving from the preferred preparatory posture toward low balls, the goalkeepers started the dive by increasing their SW (Figure 2) with a small contralateral sidestep in the direction opposite to the dive and an ipsilateral sidestep in the direction of the dive. For high balls the increase in SW was usually achieved by performing only an ipsilateral sidestep in the direction of the dive. The resultant increase in SW reached a maximum, between the contralateral and ipsilateral peak forces, that was similar in magnitude (~90% leg length) and timing (~0.72 s after light signal) for PT1, PT2, and SW75. This was in accordance with findings of Ibrahim et al. (2019), where goalkeepers stepped sideways and increased their SW from 33 to 83% of leg length. Starting from a SW narrower than SW75 takes more time for sidestepping and less time for application of force during the ipsilateral and contralateral push-off. Also, it is worth noting that when the goalkeepers started from SW100, they decreased SW to transfer from contralateral to ipsilateral push-off (Figure 2). Thus, during SW100, goalkeepers did not benefit from any horizontal CoM velocity toward the goal, as there was no sidestep to increase SW. Therefore, a SW wider than 75% of leg length does not seem to further improve the diving save performance.

Goalkeepers appeared to be more explosive in their push-offs during SW75. During high dives, the CoM traveled over significantly larger horizontal and vertical distances between CPF and IPF in SW75 than in PT1 and PT2. There was also a significant increase, during low dives, in the horizontal distance traveled by the CoM from dive onset to CPF, for SW75 compared to PT1 and PT2. This difference between preparatory postures occurred earlier during low dives than high dives, which might be because the contralateral leg contributes more to the horizontal CoM velocity during low dives than during high dives (Ibrahim et al., 2019). The goalkeepers were also more efficient during SW75 as there was significantly less countermovement during high dives and a smaller CoM vertical velocity range during low dives.

This is not the first study to find a characteristic of a sports movement in elite athletes to be less than optimal. Estevan et al. (2013) analyzed the mechanics of the taekwondo roundhouse kick starting from three different stance positions (0, 45, 90°). They found that the kick was performed with shorter reaction and execution times when starting from a 0 and 45° stance positions, rather than starting from a 90° position which was more frequently adopted by coaches and athletes. Furthermore, Ball and Giblin (2009) studied the effect of SW in the starting position on movement time of field hockey goalkeeper corner saves. They found that a SW of 1.1 m, which was wider than their preferred SW, resulted the shortest movement time.

Typical ball speed during a football penalty shot is about 20.83 m·s^−1^ (Kuhn, 1988; Morris and Burwitz, 1989; Morya et al., 2003). The practical relevance of the relatively small time difference between SW75 and the average of PT1 and PT2 that we found (0.021 s) can be translated into the distance traveled by the ball and by how much further the goalkeeper can reach during that time. A ball traveling at 20.83 m·s^−1^ will be around 50 cm closer to the goal, during preferred posture conditions than SW75 when reaching the hand position of ball contact in the current setup. Furthermore, at the same point in time (instant of ball contact) the goalkeeper can reach about 15 cm further during SW75 than in preferred posture conditions. One could argue that a faster movement would allow for the same reach, while starting the movement a little later, which would probably increase the chance of diving to the position that the ball is heading to. This is important, for instance, in a penalty shot as the time the ball takes to reach the goal (~600 ms; McMorris et al., 1993) is shorter than the movement time of goalkeepers' dive (~1,000 ms; Ibrahim et al., 2019). Additionally, if diving from SW75 will allow the goalkeeper to be 15 cm further (at the same point in time), it is possible that the goalkeeper would overreach the ball. However, overreaching will usually lead to blocking the ball with the arms or upper body instead of the hands, which is preferred over not blocking the ball.

The characteristics of the optimal preparatory posture, for an explosive sport skill, are utilized by technical and S&C coaches to make exercises, on the field and in the gym, more specific to the sport (Sheppard et al., 2016). Therefore, it might be beneficial to also perform S&C exercises, as well as technical skill training from a SW equal to 75% of personal leg length. Future studies are needed to test this hypothesis. Additionally, as the SW75 had not been trained in the present study, it is plausible that diving save performance differences with the preferred posture can be further improved after training. Therefore, we recommend performing an intervention study to train the goalkeeper physically and technically on the diving save while starting from a SW equal to 75% of personal leg length. Such studies could aim to find out how far performance improves after that the goalkeepers actually train to dive from this unaccustomed preparatory posture. Nevertheless, these recommendations need to be handled carefully due to the limitations of the current study. While the consistency of our findings over variables does suggest that the sample size was adequate to obtain sufficient power, verification of the findings in other populations is needed for a definite generalization of the results. A second limitation of this study is the ecological validity of the test used to measure goalkeepers' performance. Real game situations for goalkeepers do not only include pure diving saves from a certain preparatory posture, and movement time is not the only variable determining performance of goalkeepers. However, we believe that the test used in this study is close to real game situations in set plays (e.g., penalty shots, direct free kicks) and some open play situations, where goalkeepers have enough time to set themselves in a preparatory posture before reacting and performing a diving save. In addition, the stationary ball provided us with the advantage of accurately addressing timing parameters. With a variably incoming ball, even by using a ball canon, addressing timing parameters would be more difficult.

In conclusion, elite football goalkeepers start the diving save using a preparatory posture that is sub-optimal for performance. Preparing for the dive with a SW equal to 75% of individual leg length, which is wider than preferred, was optimal for all diving save directions and heights. When starting from this posture, goalkeepers performed better at contralateral push-off, as the average CoM velocity was faster at contralateral push-off. The goalkeepers were also more efficient in starting the dive, as smaller countermovement and vertical CoM velocity ranges occurred, before CPF, than in dives from their preferred posture.

## Data Availability

The datasets for this manuscript are not publicly available because privacy reasons: individual data could potentially be traced back to individual goalkeepers. Requests to access the datasets should be directed to rony.r.ibrahim@gmail.com.

## Ethics Statement

This study was carried out in accordance with the recommendations of name of guidelines, name of committee with written informed consent from all subjects. All subjects gave written informed consent in accordance with the Declaration of Helsinki. The protocol was approved by The Ethics Committee (VCWE) of the Faculty of Behavioral and Movement Sciences of the Vrije Universiteit Amsterdam.

## Author Contributions

RI contributed to the whole study (literature review, research design, experimental set-up, measurements, data processing, data analysis, and writing the manuscript). IK contributed to the research design, experimental set-up, data analysis, and writing the manuscript. VdB contributed to the research design, experimental set-up, and data processing. GF contributed to the research design and experimental set-up. JvD contributed to the data analysis and writing the manuscript.

### Conflict of Interest Statement

The authors declare that the research was conducted in the absence of any commercial or financial relationships that could be construed as a potential conflict of interest.
